# Increasing efficiency in the ACR Digital Mammography QC Manual's DBT Z‐resolution evaluation

**DOI:** 10.1002/acm2.70619

**Published:** 2026-05-16

**Authors:** Ryan F. Fisher, Nicholas B. Bevins, Katie W. Hulme, Colin Schaeffer

**Affiliations:** ^1^ Department of Radiology Case Western Reserve University/The MetroHealth System Cleveland Ohio USA; ^2^ Department of Radiology MaineHealth Maine Medical Center Portland Maine USA; ^3^ Imaging Institute Cleveland Clinic Cleveland Ohio USA; ^4^ Department of Diagnostic Radiology Oregon Health & Science University Portland Oregon USA

**Keywords:** mammography, quality control

## Abstract

**Background:**

The Z‐resolution evaluation in the ACR Digital Mammography (DM) QC manual measures a pseudo full width half max (pFWHM) from speck group signals through slices of the phantom for comparison to a baseline value.

**Purpose:**

The evaluation requires measurement of seven ROIs on up to nine slices, for a potential of up to 63 ROIs. This work evaluates whether measuring fewer ROIs would produce similar results.

**Methods:**

Z‐resolution data from 468 surveys from 147 mammography units were retrospectively analyzed. The pFWHM was calculated using the 6‐speck mean (pFWHM_6_) as described in the ACR manual, as well as using each single speck (pFWHM_1_), and all combinations of a 2‐speck mean (pFWHM_2_) using the center and each peripheral speck. Deviation from the respective initial measured pFWHM_initial_ was determined for all units with at least two surveys.

**Results:**

The pFWHM_1_ had an average absolute error of 6.0% relative to pFWHM_6_ across all surveys, compared to 3.9% for pFWHM_2_. For the 111 units with longitudinal data, the mean absolute deviation from pFWHM_initial_ was 7.8% for pFWHM_1_, compared to 5.6% for pFWHM_2_, and 5.1% for pFWHM_6_. For longitudinal testing, pFWHM_6_ produced two failures that were related to major component replacement. Both pFWHM_1_ and pFWHM_2_ methods caught those failures for a false negative rate of 0%. However, pFWHM_1_ had a false positive rate of 12.9%, while pFWHM_2_’s was only 2.8%. No positive test from any method was attributable to actual equipment failure.

**Conclusion:**

A single‐speck pFWHM for ACR DM QC resulted in high variability and frequent unit failures for the Z‐resolution evaluation. However, a 2‐speck pFWHM produced results clinically equivalent to the 6‐speck method. Using the 2‐speck method as an alternative methodology for Z‐resolution evaluation achieves similar results with 57% fewer measurements, increasing the efficiency of testing.

## 1 INTRODUCTION

The 2^nd^ edition of the American College of Radiology (ACR) Digital Mammography (DM) Quality Control (QC) Manual includes a digital breast tomosynthesis (DBT) Z‐resolution evaluation that measures through‐plane resolution and serves “to ensure that blurring in the Z‐direction is not excessive.”^1^ This test involves regions of interest (ROIs) drawn over each speck in the largest group in a tomographic reconstruction of the ACR DM phantom, in a method similar to the artifact spread function (ASF) described by Wu et al^2^ and summarized by Sechopoulos.^3^ Further discussion of the ASF for various commercially available phantoms on a broad range of equipment models can be found in Dalmonte et al.^4^

**FIGURE 1:**
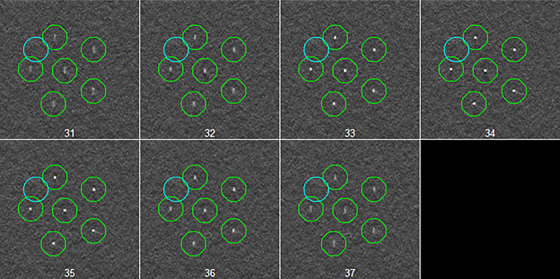
ROI measurements drawn on the six speck objects and background across seven tomographic slices of the ACR DM Phantom for the Z resolution evaluation.

**FIGURE 2:**
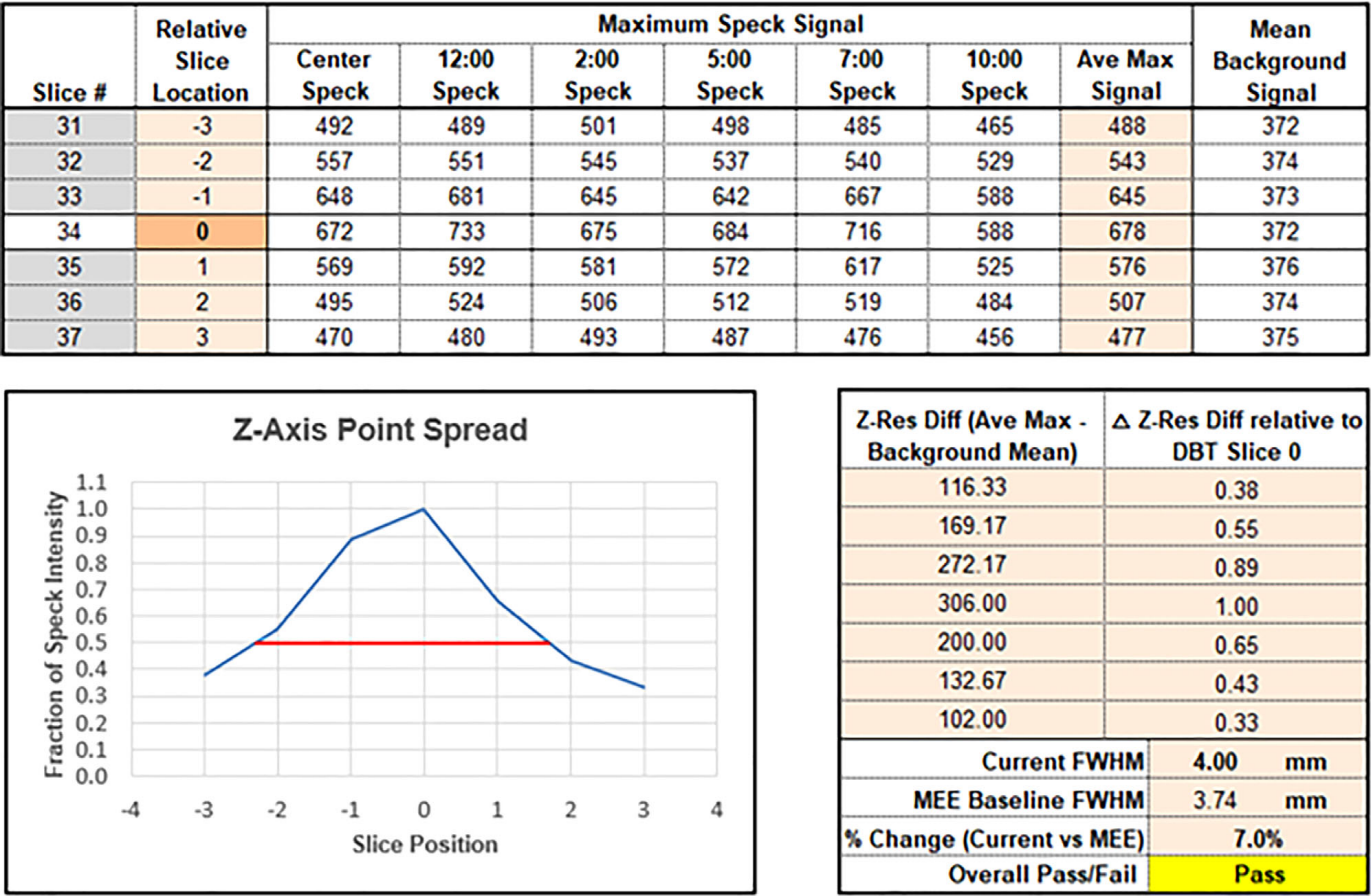
ACR Z resolution evaluation template. In the top portion, maximum speck signal values from each ROI are entered into the spreadsheet, along with the mean background signal. That data is used to calculate the Z‐res difference for each slice relative to the central slice (slice #34 here, or the 0 relative slice location), plot the Z‐axis point spread, and calculate the spread's FWHM.

The ACR DM QC manual's Z‐resolution methodology is shown in Figures 1 and 2. Figure 1 shows a close‐up of the largest speck group in the ACR's full field digital mammography (FFDM) phantom taken from seven contiguous DBT slices. The numbers at the bottom of each image represent the slice number, with slice 34 corresponding to the plane of the speck group in the phantom, as the speck objects appear brightest in that slice as speck signal decreases visibly in the slices above and below. ROIs are drawn around each of the 6 speck objects in each slice, as well as a background ROI between the specks in the 9 o'clock and 12 o'clock positions. Figure 2 is a section of the ACR's data entry spreadsheet filled out for the Z‐resolution test. The maximum pixel value from each of the 6 speck ROIs, as well as the mean background signal, is entered into the corresponding cell. The template averages the maximum signal values from each of the specks in a given slice, and then subtracts the mean background signal value to produce the signal difference for each slice, listed as “Z‐res Diff” in Figure 2, and the relative change in brightness from the maximum signal slice, “delta Z‐res Diff relative to DBT Slice 0”, which is used to plot the Z‐axis Point Spread and calculate a pseudo full width half max (pFWHM) that characterizes the fraction of the maximum speck intensity visible in the surrounding slices.

In clinical practice, an initial pFWHM is set as a baseline value at acceptance testing or during a mammography equipment evaluation (MEE) after a major component replacement, as the length of pFWHM can be affected by changes in system components such as newly installed tubes, detectors, generators, etc. During subsequent annual testing, the Z‐resolution evaluation is repeated and the calculated pFWHM is compared to the MEE value. An absolute deviation in measured pFWHM of greater than 30% constitutes a failure. Practically, ± 30% is a very wide range and the Z‐resolution test almost never fails on annual testing. In Figure 2, the pFWHM is calculated as 4.00 mm, which is 7% larger than the 3.74 mm baseline pFWHM measured during the equipment's initial mammography equipment evaluation (MEE).

**FIGURE 3:**
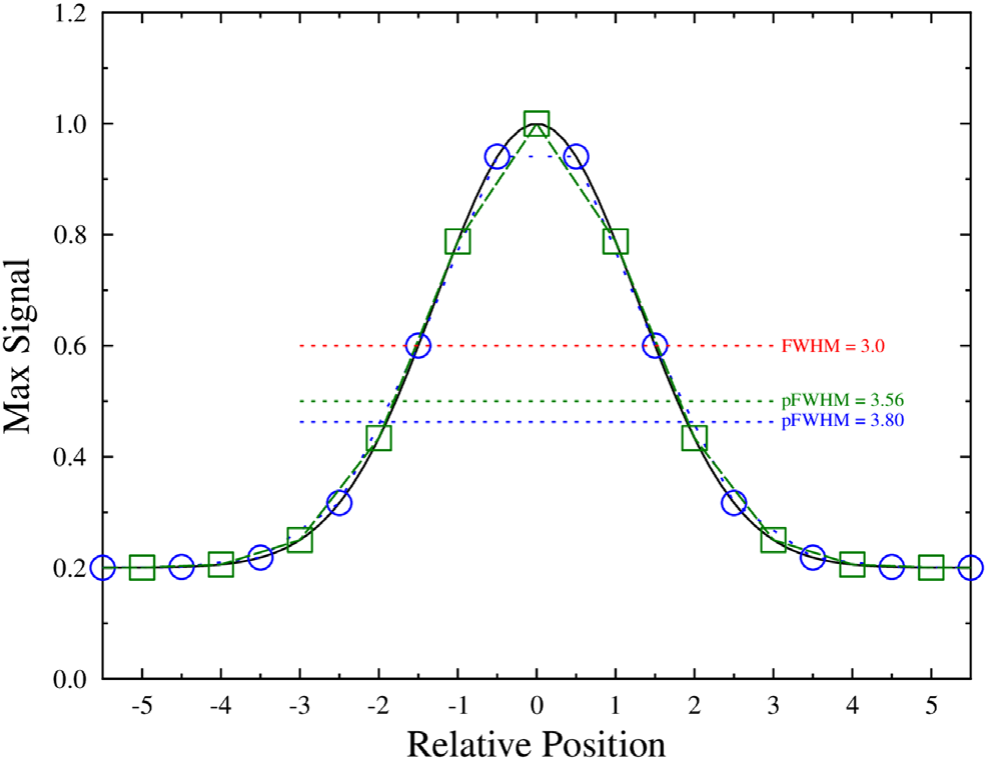
Theoretical Gaussian distribution showing both the effect of the ACR's FWHM formalism producing broader pseudo FWHMs of 3.56 and 3.8 mm compared to a true FWHM of 3.0 mm. The two pFWHMs illustrate the increased width produced when the maximum signal value is split between two slices rather than centered at relative position 0.

This paper distinguishes the pFWHM from a true full width half maximum measurement of the Z‐axis point spread because the formalism used by the ACR DM manual does not account for a baseline offset in the calculation because it uses the background mean values, rather than the background maximum values, to determine the relative speck intensity. Farther from the central plane, the maximum pixel value within an ROI falls off as less of the speck group is included, but the ROI value converges to the background maximum value, not the background mean value. Since the maximum pixel value is larger than the mean pixel value, the baseline of the FWHM curve never reaches zero, resulting in an artificially broadened Z‐resolution measurement compared to a true FWHM measurement. This is illustrated in Figure 3, where a true FWHM, without the background offset, of 3.0 mm is shown compared to the broader 3.56 mm and 3.80 mm pFWHMs calculated according to the ACR's method.

The pFWHM length is a function of the system's acquisition geometry, inherent reconstruction algorithm, and the location of the speck group within the reconstructed slices. The most common failure not related to the recent replacement of major system components occurs when the physical location of the speck group in the phantom straddles two reconstructed slices. Because both slices that the speck group straddles have similar maximum pixel values, this “split slice” situation results in a reduction in the overall maximum peak pixel value and the broadening of the pFWHM peak, as seen in a theoretical Gaussian model in Figure 3. In clinical data, the point spread profile frequently deviates from a pure Gaussian function and this scenario can cause the calculated pFWHM to extend beyond 30% of the baseline FWHM without any deficiencies in the equipment. In these cases, the remedy is to uncompress the phantom, recompress, and reimage the phantom, hopefully shifting the speck plane into a single slice plane and producing a passing test result.

The ACR DBT Z‐resolution test can be very time‐consuming, as up to 63 ROIs must be drawn and measured, depending on the number of adjacent slices required to produce the pFWHM. This retrospective study evaluates whether measuring fewer ROIs would produce similar clinical results by analyzing data collected from four institutions.

## 2 METHODS

Test data from the ACR DM manual's Z‐resolution test from 468 mammography surveys of 147 individual mammography units across four institutions were retrospectively analyzed. Surveys included a mix of MEEs and annual surveys. MEE status was not differentiated in the initial analysis, but was reviewed in investigating potential test failures. No retrospective analysis of phantom images was conducted in this study. Speck ROI measurements collected by various physicists in existing survey reports were used to calculate pFWHM values using various combinations of speck signals. Eighty unique FFDM phantoms were used in the acquisition of survey data, though for any given mammography unit the same phantom was used across all surveys, as is generally the case in clinical practice. Manufacturer and model information for the mammographic units included in this study are listed in Table 1.

**TABLE 1:** Absolute deviations from the gold standard for pFWHM values calculated using one and two specks.

			pFWHM_1_ deviation from pFWHM_6_ ^*^			pFWHM_2_ deviation from pFWHM_6_ ^**^		
	Units	Surveys	Mean	Max	SD	Mean	Max	SD
Overall	147	468	6.0%	47.8%	5.6%	3.9%	28.2%	3.6%
Hologic	53	162	6.7%	32.2%	5.5%	4.3%	24.8%	3.5%
Siemens	85	264	5.3%	37.8%	5.1%	3.6%	23.8%	3.5%
GE Healthcare	9	42	7.9%	47.8%	7.5%	5.0%	28.2%	4.0%
3Dimensions	19	58	5.3%	19.1%	4.1%	3.3%	13.9%	2.6%
Selenia Dimensions	34	104	7.5%	32.2%	6.1%	4.8%	24.8%	3.8%
Mammomat Inspiration	38	150	5.8%	27.5%	4.7%	3.9%	23.8%	3.3%
Mammomat Inspiration Prime	11	45	5.8%	37.8%	5.1%	4.0%	23.2%	3.6%
Mammomat Revelation	35	69	4.0%	28.0%	5.7%	2.6%	20.9%	3.5%
Senographe Essential with Senoclaire	6	31	7.9%	47.8%	7.2%	5.0%	19.8%	4.0%
Senographe Pristina	3	11	7.4%	40.5%	7.3%	4.9%	21.9%	4.0%

^*^Values are across all six individual specks.

^**^Values are across all five center + peripheral speck combinations.

Visual Basic for Applications (VBA) scripts were used to extract the relevant ROI speck value measurements from existing survey spreadsheets, which were all based on the standard ACR provided template. VBA scripts were also used to calculate various pFWHM values for each survey. The pFWHM calculated using all six specks, further designated as pFWHM_6_, was treated as the gold standard for comparison to other measurements. Alternate pFWHM values were calculated using each single individual speck in the phantom, designated as pFWHM_1_. Additionally, two‐speck pFWHMs, pFWHM_2_, were calculated using all combinations of the center and a single peripheral speck from each survey. The number of slices collected for each pFWHM calculation matched the original physicist testing data and ranged from 5 to 9. Statistical analysis was performed to evaluate differences between pFWHM_1_ and pFWHM_2_ compared to pFWHM_6._ Difference scores were calculated by subtracting pFWHM_6_ values from each of the other two test methods and pairwise F‐tests for equality of variance were used to compare variability across vendors within each method as well as between methods overall.

For the second part of the study, longitudinal comparison of Z‐resolution test pFWHM measurements was performed on 111 mammography units from the collected sample that had at least two surveys available. Units without multiple surveys were excluded from this portion of the study due to lack of longitudinal comparison. In total, 431 individual surveys were included in this portion. The number of surveys per mammographic unit ranged from two to nine, with an average of 3.9 surveys. For each of the 111 units with at least two surveys, a MATLAB script was created to compare each of the calculated pFWHM values from a given survey to the baseline value from the oldest available survey (pFWHM_initial_). For this study, data was not initially collected to determine if subsequent surveys were annual tests or post repair MEEs. The oldest survey available for each unit was considered the MEE, which set the baseline pFWHM_initial_ value that all subsequent pFWHM measurements are compared to for the purposes of determining if the Z‐resolution test passed. Separate pFWHM_initial_ values were used for each of the individual pFWHM_1_, pFWHM_2_, and pFWHM_6_ metrics investigated. Surveys where a pFWHM value exceeded 30% of pFWHM_initial_ were marked as failing. Using the pFWHM_6_ as the gold standard, false positive and false negative rates were calculated for both the pFWHM_1_ and pFWHM_2_ methods. A false positive was defined as when an abbreviated method produced a failure that pFWHM_6_ did not, and a false negative as when an abbreviated method failed to detect a failure produced by pFWHM_6_.

In normal clinical practice, an MEE for a repair such as a detector or X‐ray tube replacement would establish a new baseline pFWHM_initial_ that would apply to future surveys. Ignoring the MEE status of subsequent surveys in this study means larger variations from pFWHM_initial_ are expected, potentially leading to test failures that would not fail in actual clinical practice. Model and manufacturer names for each survey were collected to investigate potential differences between mammography systems.

## 3 RESULTS

### 3.1 Comparison to pFWHM_6_ gold standard

For each survey, pFWHM_1_ and pFWHM_2_ were compared to pFWHM_6_. Mean and maximum absolute deviation from pFWHM_6_, as well as the standard deviation for both are shown in Table 1. Overall results include all 147 mammography units, which are broken out by manufacturer and model for comparison. Results for pFWHM_1_ include calculations made on each of the six individual specks in the group, (6 measurements per survey) while results for pFWHM_2_ include all combinations of the center and a peripheral speck (five measurements per survey).

Single speck pFWHM measurements showed a substantially larger variation from pFWHM_6_ compared to using two specks, as evident in the larger mean, max, and standard deviation values in Table 1 for pFWHM_1_ compared to pFWHM_2_. The elevated maximum values seen for pFWHM_1_ reflects a broadly noisier distribution, as at the 75^th^ and 95^th^ percentiles of absolute deviation from pFWHM_6_, pFWHM_1_ produced deviations of 8.7% and 17.0% compared to 5.4% and 11% for pFWHM_2_, respectively. The reduction in mean deviation going from pFWHM_1_ to pFWHM_2_ was highly significant both overall and for every vendor and model individually (*p* < 0.001). Overall, in an F‐test for equality of variance, the 2‐speck pFWHM_2_ demonstrated a statistically significant 2.2‐fold lower variance in deviation from pFWHM_6_ compared to pFWHM_1_ (0.051 vs. 0.112; F = 2.21, *p *< 0.0001), a finding that also held true across all vendors and individual models (*p* < 0.0001).

For pFWHM_1_ measurements, a Friedman's test among each of the individual specks was highly significant (*χ* ^2^ = 18.18, df = 5, *p* = 0.003), indicating that choice of speck used significantly affects the pFWHM_1_ width. Post‐hoc pairwise Wilcoxon signed‐rank tests with Bonferroni correction identified speck 6, in the 9 o'clock position and furthest from the chest wall, as the only statistically distinct speck, showing a significant difference from the central speck (*p* = 0.0014). All other specks were statistically interchangeable. Somewhat interestingly, the 9 o'clock speck had the highest max signal intensity in 68% of the surveys, a result that held across all vendors (Hologic 62%, Siemens 71%, GE 76%).

For pFWHM_2_ measurements, a Friedman's test revealed no significant difference in pFWHM among the five different combinations of center and peripheral speck used to calculate pFWHM_2_ (*χ* ^2^ = 4.67, df = 4, *p* = 0.323), indicating that all 2‐speck combinations are statistically equal.

Paired *t*‐tests were performed to determine if the alternate pFWHM methods were statistically different from pFWHM_6_. Overall, pFWHM_1_ showed a small but statistically significant negative deviation from pFWHM_6_ (mean difference = −0.037 mm, 95% CI [−0.048, −0.027], *p* < 0.001), indicating a systematic underestimation of pFWHM compared to the gold standard. This underestimation held true across most vendors, and models with the exception of the Hologic 3Dimensions (*p* = 0.19), and GE Senographe Pristina (*p* = 0.44), which was underpowered with only 3 units.

In contrast, pFWHM_2_ measurements were statistically equivalent overall to pFWHM_6_ (mean difference = −0.001 mm, 95% CI [−0.014, +0.013], *p* = 0.905), though some specific models were significantly different, including the Hologic 3Dimensions (*p* = 0.05) and Selenia Dimensions (*p* = 0.03), and the Siemens Inspiration (*p* = 0.003). When present, these differences showed inconsistent direction and small magnitude (0.02–0.05 mm), suggesting no clinically meaningful vendor specific systematic bias as average pFWHM values for those units were on the order of 3.5–5.3 mm.

### 3.2 ACR DM QC manual Z‐resolution test performance

As previously described, the ACR specifies that a measured pFWHM that deviates by more than 30% from pFWHM_initial_ constitutes a failure for the Z‐resolution test. Table 2 shows the mean absolute deviation from pFWHM_initial_ for all three pFWHM measurement methods in the study for units that had at least two surveys. As with Table 1, overall results are shown as well as results broken out by manufacturer and model. The mean deviation results for each method include a sum of all measurements of the same type. So pFWHM_1_ includes six measurements per survey and pFWHM_2_ includes five, with each measurement compared to the baseline measured with the same speck(s).

**TABLE 2:** Absolute deviation from initial measurement for subsequent measurement of various pFWHM values & number of test failures for each method.

			Mean absolute deviation from pFWHM_initial_			Deviations > 30% from pFWHM_initial_		
	Units	Surveys	pFWHM_6_	pFWHM_2_ ^*^	pFWHM_1_ ^*^	pFWHM_6_	pFWHM_2_ ^**^	pFWHM_1_ ^**^
Overall	111	431	5.1%	5.6%	7.8%	2	4.2	10.2
Hologic	34	143	5.3%	5.2%	8.3%	0	0.6	3.2
Siemens	69	248	4.9%	5.5%	7.2%	2	2.4	4.3
GE Healthcare	8	41	6.1%	8.2%	11.0%	0	1.2	2.7
3Dimensions	12	51	4.8%	4.4%	6.0%	0	0.0	0.0
Selenia Dimensions	22	92	5.6%	5.6%	9.6%	0	0.6	3.2
Mammomat Inspiration	36	148	5.8%	6.4%	8.1%	2	2.0	3.2
Mammomat Inspiration Prime	11	45	4.0%	4.8%	7.0%	0	0.2	0.5
Mammomat Revelation	22	55	4.0%	4.4%	5.9%	0	0.2	0.7
Senographe Essential with Senoclaire	6	31	5.4%	7.7%	11.4%	0	0.4	1.8
Senographe Pristina	2	10	8.1%	9.6%	9.7%	0	0.8	0.8

^*^Mean deviation values for pFWHM_2_ and pFWHM_1_ include all individual measurements for each.

^**^Number of failures for pFWHM_2_ and pFWHM_1_ are normalized by the number of measurements per method, 5 pFWHM_2_ for and 6 for and pFWHM_1._

For mean absolute deviation from pFWHM_initial_, pFWHM_1_ produced statistically significant higher variation from pFWHM_6_, both overall and for all vendors and models (all *p* < 0.05) except for the GE Healthcare Senographe Pristina, which was underpowered at just 2 units and 10 combined surveys. The pFWHM_2_ method was statistically indistinguishable from pFWHM_6_ across all models.

The right portion of Table 2 shows instances where a follow‐up survey's pFWHM measurement for each method had an absolute deviation of greater than 30% from pFWHM_initial_, constituting a failure. Each measurement method was only compared to the baseline measured with the same speck or speck combination. For failures in the Table, results for both pFWHM_1_ and pFWHM_2_ are normalized to the number of groups analyzed for each method, or 5 for pFWHM_2_ and 6 for pFWHM_1_, for easier comparison to pFWHM_6_ since multiple pFWHMs measured per survey could potentially lead to multiple failures from a single survey, inflating the total failure count.

The ACR's pFWHM_6_ method produced only two failures across all 431 surveys and 111 units. In both of those cases, the survey producing the failure was a post repair MEE for installation of new X‐ray tube. As previously mentioned, our initial analysis did not consider MEE status of follow‐up surveys. Per the ACR DM QC manual, since changing major components can drastically change the pFWHM, a physicist would set a new baseline value in those instances. As such, neither of the pFWHM_6_ produced failures constitute “real” clinical equipment failures. Of note, for both these units (and many others in the data set), other surveys also included MEE tests but did not produce failures of the Z‐resolution test, as all FWHM values calculated in those surveys were still within 30% of the original pFWHM_initial_ even after major component replacement.

As seen in Table 2, the pFWHM_1_ method produced significantly more test failures than the pFWHM_6_ method. Specifically, 61 total failures were produced across all 6 specks, leading to a total of 43 surveys where at least one pFWHM_1_ measurement resulted in a failure. This is not surprising given the increased deviation from baseline seen in the pFWHM_1_ results. It should be noted that both failures in pFWHM_6_ were also picked up using only pFWHM_1_, leading to a false negative rate of 0%. The increased number of failures led to an overall false positive rate of 12.9%, with results varying by vendor as shown in Table 3.

**TABLE 3:** False positive and negative rates for the Z resolution test performed measuring only one or two specks.

			False positive rate		False negative rate	
	*n*	Surveys	pFWHM_2_	pFWHM_1_	pFWHM_2_	pFWHM_1_
	111	431	2.8%	12.9%	0.0%	0.0%
Hologic	34	143	1.8%	12.8%	N/A	N/A
Siemens	69	247	2.3%	9.0%	0.0%	0.0%
GE Healthcare	8	41	9.1%	33.3%	N/A	N/A
3Dimensions	12	51	0.0%	2.6%	N/A	N/A
Selenia Dimensions	22	92	2.9%	18.6%	N/A	N/A
Mammomat Inspiration	36	147	1.8%	9.1%	0.0%	0.0%
Mammomat Inspiration Prime	11	45	2.9%	8.8%	N/A	N/A
Mammomat Revelation	22	55	3.0%	9.1%	N/A	N/A
Senographe Essential with SenoClaire	6	31	8.0%	28.0%	N/A	N/A
Senographe Pristina	2	10	12.5%	50.0%	N/A	N/A

**FIGURE 4:**
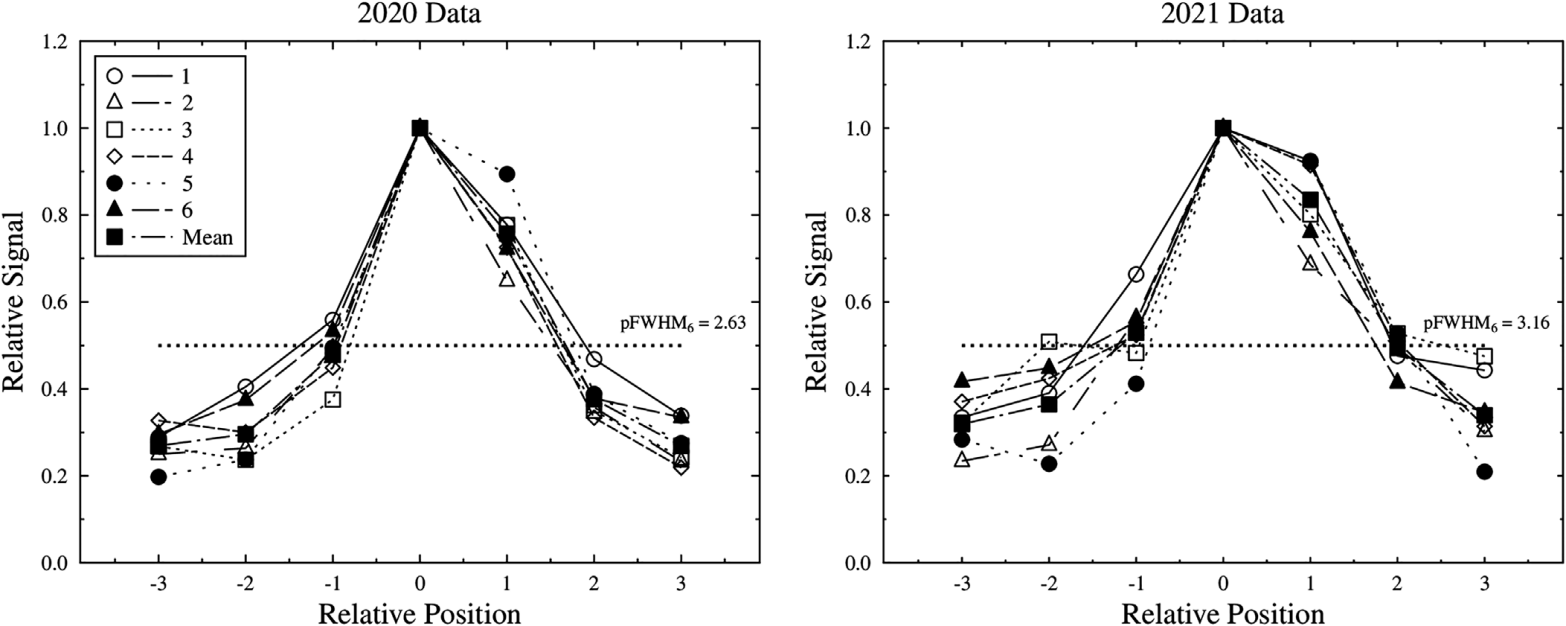
Example of a “split‐slice” failure method from clinical data for a mammograph unit over two years. The 2020 data represent pFWHM_initial_, while in 2021 the speck group was straddling two reconstructed slices, leading to a 20% increase in pFWHM_6_ and one combination of pFWHM_2_ to exceed the 30% failure criteria.

**FIGURE 5:**
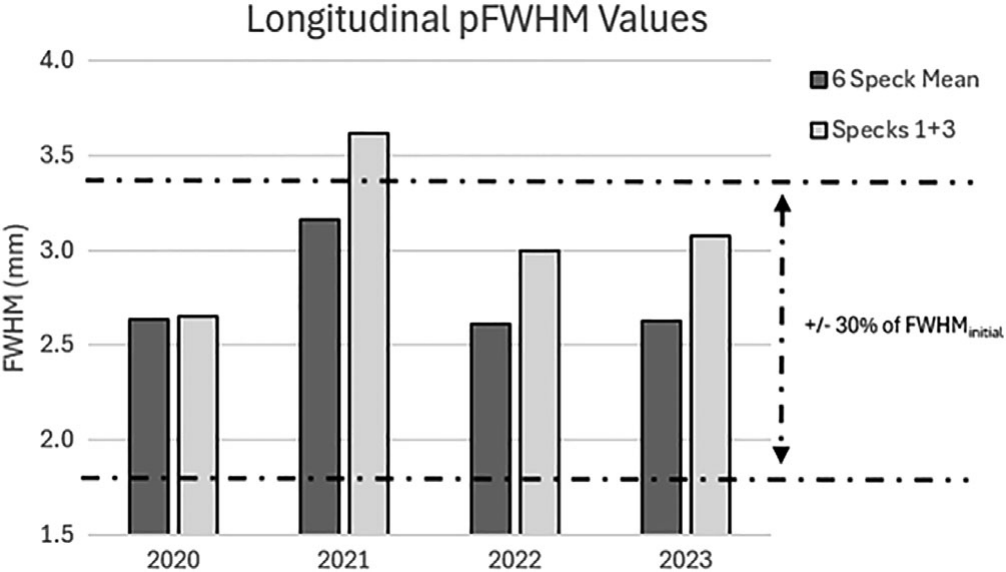
Annual test pFWHM values from the unit in Figure 4 that produced a failure with pFWHM_2_. Both pFWHM methods produced higher measurements in 2021 due to a split‐slice scenario, but only pFWHM_2_ triggered a failure. Both methods dropped to passing in subsequent years.

For the pFWHM_2_ method, again, both the pFWHM_6_ failures were identified, leading to a false negative rate of 0%. Additionally, several surveys triggered failures that pFWHM_6_ did not, with an overall false positive rate of 2.8%. Investigation of false positive surveys showed that they were broadly the result of either of two scenarios:

1) Major component replacement with MEE baseline reset– In these cases, a major component had been replaced at some point in the unit's history, which in clinical practice would have resulted in the resetting of the baseline value. In a representative example, on a survey following a tube replacement, the pFWHM_6_ deviation from pFWHM_initial_, if ignoring the replacement and comparing to the first available survey, was just below the failure threshold at 28%, compared with 31% for pFWHM_2_. The large deviation in both measures was the result of the new tube and in clinical practice a new pFWHM_initial_ would have been established during an MEE. As such, for this study, major component replacement related Z‐resolution failures do not represent actual clinical test failures.
2) Split‐slice artifact—As described in the introduction, in these cases the physical speck objects are located between two reconstructed slices, spreading the pFWHM peak, possibly to the point of causing a test failure. A representative example of a split slice failure is shown in Figure 4, from a GE Senographe Essential w/ SenoClaire. The Z‐axis point spread is shown from surveys in 2020 and 2021. Both surveys were annual evaluations and no major system components were changed in between the two measurements. In both images, relative intensity values for each speck individually, as well as the mean from all six specks is shown. In 2020, the center slice at the “0” relative position is the maximum value by far for all specks, which leads to a pFWHM_6_ of 2.63 mm. The following year, relative values of specks in the “+1” position are almost universally higher, as the physical location of the speck in the phantom is closer to the +1 slice than it was in 2020. This has the effect of both spreading the peak of the point spread and raising the relative value of the tails at the ± 3 slice positions. These effects combine to result in a pFWHM_6_ of 3.16 mm, which is 20% higher than the 2020 value. While the pFWHM_6_ measurement from 2021 is still within 30% of the 2020 value, pFWHM_2_ values calculated with specks 1+3 are 36.5% larger than its 2020 value. Additionally, calculated pFWHM_2_ with specks 1+4 was 28.9% higher. For this particular unit, nothing was wrong with the equipment but due to the relative position of the specks within the reconstructed slices both pFWHM_6_ and pFWHM_2_ were elevated in 2021, though only pFWHM_2_ triggered a failure. Of note, in the annual surveys for the years following the 2021 data, both pFWHM_2_ and pFWHM_6_ dropped to within passing values, as shown in Figure 5. Split‐slice situations are not uncommon, though they rarely trigger failures of the Z‐resolution test. In the event of a failure, uncompressing the phantom, recompressing, and imaging the phantom will often reposition the speck group within the reconstructed slices and produce passing results. Split‐slice artifact related failures also do not represent true equipment failures, as they can be easily remedied by reimaging the phantom.

In total, pFWHM_2_ produced 11 total survey failures, defined as when any single 2‐speck combination resulted in failure. These surveys included the two MEE related failures produced by the pFWHM_6_ method. Of the nine other failures, five were related to MEE tests where a new baseline would have been established during the unit's test history, eliminating the failure. In these five cases, pFWHM_6_ measurements led to variation just below 30% from pFWHM_initial_. The four other failures were all produced by the “split‐slice” scenario. In those instances, one of which is further detailed in the discussion, the pFWHM_6_ method led to inflated % differences from pFWHM_initial_ on the order of 11%–20% due to the peak spread. For each of these units, at least one of the 2‐speck calculation methods produced a deviation >30%. In three cases, only one combination of the center and a peripheral speck produced a failure, while in the fourth case, three different combinations produced a failure. Of the four “split‐slice” related failures produced by pFWHM_2_, two had subsequent surveys available after the split‐slice survey year. In both cases, both the pFWHM_6_ and pFWHM_2_ measurements dropped back to within a few % of pFWHM_initial_. In the other two cases, the failing split‐slice test was the most recent survey available. Although all 2‐speck combinations produce similar pFWHM_2_ measurements from a statistical standpoint, empirically, a combination of specks 1+2, the center and 12 o'clock specks, produced the fewest failures, while a combination of speck 1+6, the center and 9 o'clock, produced the most.

Of note, all the measurements in this paper were duplicated with pFWHM_2_ calculated using all combinations of 2 peripheral specks in the group, but results were largely similar to those from the center plus a peripheral speck, so they were omitted for clarity.

## 4 DISCUSSION

The measurement of signal from all 6 speck objects in the ACR's Z‐resolution test helps to effectively average out any possible signal differences related to variation in the physical phantom speck objects, as well as any differences resulting from the speck group not being perfectly in‐plane with the reconstructed slices. Measuring a pFWHM using only a single speck instead of all six produced a small but statistically significant reduction in pFWHM measurements compared to the gold standard, an effect likely due to no longer averaging planar effects across the speck group. While the mean overall magnitude of variation from pFWHM_6_ was low, pFWHM_1_ also produced much noisier results, with large maximum deviations from the gold standard. In clinical practice, this led to an unacceptably large number of false positive failures of the Z‐resolution test, as the pFWHM_1_ from a single speck is much more likely to deviate by >30% from pFWHM_initial_ than pFWHM_6_.

After the failure of pFWHM_1_ as an adequate replacement for the ACR's method, the use of 2 specks was explored. As shown in Table 1, pFWHM_2_ moving to using a combination of the center speck and a peripheral speck resulted in substantially better agreement with pFWHM_6_, and a statistically significant reduction in both absolute deviation and variance from the baseline metric. More importantly, pFWHM_2_ produced clinical results for the Z‐resolution test much more in‐line with the ACR's method. As shown in Table 3, measuring pFWHM_2_ produced a false negative rate of 0% and reduced the false positive rate to 2.8% across all units. As discussed, failures triggered using pFWHM_2_ were either the result of an MEE or a split‐slice scenario.

While the overall false positive rate of the Z‐resolution test using pFWHM_2_ was already low, of the 9 total test failures produced, none are the result of actual equipment failures, and all could be remedied by either resetting pFWHM_initial_ values during an MEE evaluation or re‐imaging the phantom to recenter the speck group. As such, for a practicing physicist, using pFWHM_2_ produces clinically equivalent test results to the ACR specified pFWHM_6_ method while saving considerable time by reducing the number of drawn ROIs. The large number of surveys across multiple institutions, vendors, and models, including the use of over 80 unique ACR FFDM phantoms, point toward the robustness of the two‐speck method for pFWHM measurement.

The choice of which two specks to use is not statistically significant, though empirically in this study specks 1+2 produced the fewest failures, while specks 1+6 produced the most. Of note is that in the one‐speck analysis, speck 6 was the one speck that was statistically different from the others, as well as very often the speck with the highest center slice signal. This is possibly due to its position farthest from the chest wall, which can negatively affect spatial resolution. Meanwhile, speck 2 is closest to the center of the image, where spatial resolution is greater due to focal spot effects, which possibly explains why the speck 1+2 combination empirically produced the fewest failures.

## 5 CONCLUSION

To any physicist drawing 63 ROIs who ever wondered if just measuring a single speck would produce the same test results, the answer is sadly, “no.” But for measurement of DBT through plane resolution using the ACR's Z‐resolution test, this work demonstrates that pFWHM measurements made using ROIs from only two specks would have produced clinically equivalent longitudinal results to those made using ROIs from all specks for almost all of 431 surveys of 111 units. In the few instances where measuring two specks produced a failure due to a split‐slice scenario, it can be remedied by reimaging the phantom. These results held true across all vendors and models of mammography equipment tested, using 80 unique ACR phantoms. Changing from seven total ROI measurements per slice to three could reduce the number of measurements by 57%, thereby saving time and effort while providing the same test result. While some physicists, including some of the authors of this paper, are using automated methods for calculating pFWHM by exporting phantom slice images and running scripts to extract ROI values, this can add complexity to annual testing and a large number of practicing physicists are manually drawing ROIs on the acquisition workstation. It is these physicists who would be served by the streamlining of the Z‐resolution test.

Since MEEs were not originally considered in the longitudinal analysis here, even when major components were replaced the pFWHM_2_ method had a false positive rate of less than 3%. In addition, the authors would like to note that the absence of any non‐MEE related failures using pFWHM_6_ over 431 surveys demonstrates the stability of this metric over time, and that it may be of limited utility for ongoing QC and be more useful for troubleshooting than a requirement for annual surveys.

While the ACR QC Manual specifies that the Z‐resolution test be performed using the average of six speck ROIs, FDA requirements under MQSA require that medical physicists follow a quality assurance program that is “substantially the same as the quality assurance program recommended by the image receptor manufacturer.” Whether calculating Z‐resolution using only two specks counts as “substantially similar” might depend on the individual regulator, so physicists should be cautioned to ask. The authors feel that the 0% false negative rate of the 2‐speck calculation, combined with a very low false positive rate that is easily mitigated by reshooting a phantom show that the method is catching any clinically meaningful failures. Even if regulators do not accept the 2‐speck method of testing, the authors hope that those involved in writing the ACR's DM Quality Control Manual will consider these results in future updates to help streamline testing.

## AUTHOR CONTRIBUTIONS

All authors significantly contributed to the data collection and analysis, as well as the writing of this manuscript.

## CONFLICT OF INTEREST STATEMENT

The authors declare no conflicts of interest.
